# The dynamics of biofilm development and dispersal should be taken into account when quantifying biofilm via the crystal violet microtiter plate assay

**DOI:** 10.1016/j.bioflm.2024.100207

**Published:** 2024-06-20

**Authors:** Jens Bo Andersen, Morten Rybtke, Tim Tolker-Nielsen

**Affiliations:** Costerton Biofilm Center, Department of Immunology and Microbiology, University of Copenhagen, DK-2200, Copenhagen, Denmark

## Abstract

The crystal violet microtiter plate biofilm assay is often used to compare the amount of biofilm formed by a mutant versus wild-type or a compound-treated biofilm versus the non-treatment control. In many of these studies the amount of biofilm is assessed only at one single time point. However, if the dynamics of biofilm development of the mutant (or compound-treated biofilm) is different than that of the wild-type (or non-treatment control), then biofilm quantification at a single time point may give misleading results. To overcome this shortcoming of the common biofilm quantification technique, we recommend to use a serial dilution-based crystal violet microtiter plate biofilm assay for easy assessment of the dynamics of biofilm development and dispersal. We demonstrate that the dilution-resolved crystal violet assay displays the dynamics of *Pseudomonas aeruginosa* biofilm development and dispersal as efficient as a time-resolved crystal violet assay. In addition, focusing on mutants of different parts of the c-di-GMP signaling system in *P. aeruginosa*, we provide an example illustrating the need to assess biofilm dynamics instead of quantifying biofilm biomass at a single time point.

## 1 Introduction

The crystal violet microtiter plate biofilm assay was developed in the 1980s by Christensen and coworkers [1], and it was popularized in the mid-to-late 1990s by Mack and coworkers [2] and O'Toole and Kolter [3,4], and has been used ever since in countless biofilm studies. In this assay, the microbes are grown in the wells of microtiter trays, and the amount of biofilm formed on the well surfaces is subsequently quantified by removal of the liquid medium, followed by staining of the biofilms with crystal violet, solubilisation of the bound crystal violet, and quantification by absorbance measurements. The assay is often used to study biofilm formation of mutants compared to wild-type, or to study the effect of chemical compounds on biofilm formation compared to untreated controls. In many of these studies the amount of biofilm is quantified at a single time point after a certain time period of biofilm formation. However, here we argue that the dynamics of biofilm development and dispersal should be taken into account when quantifying biofilm via the crystal violet microtiter plate assay.

**Fig. 1:**
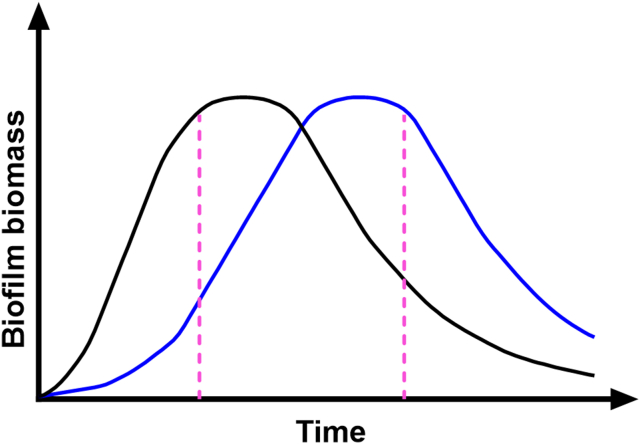
Generic plot visualizing the problem of biofilm quantification at a single time point. The figure shows the kinetics of biofilm development and dispersal of a wild-type (black curve) and a mutant (blue curve) with delayed biofilm formation. Choosing only a single time point for measurement (exemplified by dashed pink lines) may lead to wrong conclusions. (For interpretation of the references to colour in this figure legend, the reader is referred to the Web version of this article.)

Microbes generally form biofilm as long as the conditions are adequate, and transition to a planktonic state via a dispersal process when the conditions are unfavorable for biofilm formation [5], [[6], [7], [8], [9], [10], [11]]. The biofilm dispersal process can, depending on the microbial species, be triggered by different factors such as nutrient or oxygen limitation, or the accumulation of waste products or signal molecules [5], [[6], [7], [8], [9], [10], [11]]. Accordingly, when biofilm development occurs in the wells of microtiter trays the biofilm biomass will build up for a certain time period and will then decline as the biofilms disperse. Because of the dynamics of biofilm development the amount of biofilm obtained from a crystal violet microtiter plate assay will be highly dependent on the sampling time point. Thus, if biofilm formation of a mutant and wild-type (or a compound-treated and non-treated control) are compared at a single time point, the results can be misleading if the mutation (or compound) affects the dynamics of biofilm formation (Fig. 1). To mitigate this problem, a method that displays the dynamics of biofilm formation and dispersal should be used.

Time-course studies of biofilm growth in microtiter plates have occasionally been done with inoculation and independent processing of replicate plates for each time point, e.g. Ref. [6]. However, this procedure is laborious and inaccurate due to considerable plate-to-plate variation. Govantes and co-workers developed a serial dilution-based crystal violet microtiter plate biofilm assay in which the microbes are serially diluted in the wells of a microtiter plate, and incubated for a defined period of time prior to quantification of the amount of biofilm in the wells [12]. The method is based on the use of serially diluted inocula resulting in biofilms whose development is shifted in time so that a plot of the biofilm biomass as a function of inoculum size resembles a plot of the biofilm biomass as a function of time. Indeed, Govantes developed a biofilm growth curve synchronization program that provides a time-shift coefficient enabling the conversion of biofilm versus inoculum size plots into fictive biofilm versus time plots [13]. The Govantes group has used the dilution-based method for studying mechanisms of biofilm formation and dispersal in *Pseudomonas putida* and *Pseudomonas syringae* [[14], [15], [16], [17]]. In addition, we have used the method for studying biofilm dispersal in *P. aeruginosa* [18], and Gahlot et al [19] have used the method for studying mechanisms of biofilm formation in *Yersinia pseudotuberculosis* [19]. However, the dilution-based method has not been widely adopted by the biofilm research community. We believe this is due to the fact that the shortcoming of the crystal violet assay and the strategy to overcome it have not been directly addressed in any of the previous studies. Accordingly, the aim of the present short communication is to emphasize the need for using the dilution-based method in studies where microtiter plate-grown biofilms are quantified.

## 2 Materials and methods

### 2.1 Bacterial strains and growth media

The *Pseudomonas aeruginosa* PAO1 strain [20] and derived isogenic mutant strains *bifA* [21] and *dipA* (this study) were used in this study. The *dipA* mutant was constructed in this study according to the procedure of [22]. The growth media used were Lysogeny broth (LB) [23] and AB medium supplemented with trace minerals, 0.2 % glucose, 0.5 % Casa amino acids and 1 μM FeCl_3_ [24].

### 2.2 Crystal violet microtiter plate biofilm assay

The crystal violet microtiter plate biofilm assay was done as described by O'Toole and Kolter [4] with modifications. Briefly, a 20 h old culture of *P. aeruginosa* PAO1 (cultivated at 37 °C in LB or AB medium supplemented with trace minerals, 0.2 % glucose, 0.5 % Casa amino acids and 1 μM FeCl_3_) was diluted into the same growth medium to create an inoculation culture with an optical density at 600 nm of 0.1 (corresponding to 1.2 × 10^8^ CFU/ml). Then 100 μl aliquots of the inoculation cultures were transferred to the wells of microtiter plates (Tissue Culture Testplate 96F, Techno Plastic Products AG, Switzerland). Subsequently, the plates were sealed with an air-permeable lid (Sandwich cover CR1596, Enzyscreen, The Netherlands) and were incubated on a rotary shaker (Model HS501 digital, IKA, Germany) at 37 °C with 200 RPM. Following 2, 4, 6, 8, 10 and 12 h of incubation, a plate was withdrawn from incubation, the culture supernatants were discarded, and the amounts of biofilm present in the wells were quantified by crystal violet staining. To this end, the biofilms were washed three times in 280 μl of milliQ water and the microtiter plates were air dried at 37 °C for 2 h to fixate the biofilms. The biofilms were then stained using 250 μl per well of a 0.1 % (w/v) crystal violet solution for 15 min, washed 3 times with 300 μl of milliQ water, and air dried for 1 h at 37 °C. The biofilm biomass was subsequently quantified by resolubilizing the crystal violet in 300 μl of 30 % acid acetic for 10 min, followed by absorbance readings at 590 nm (A590) using a VIKTOR plate reader (PerkinElmer).

### 2.3 Serial dilution-based crystal violet microtiter plate biofilm assay

The serial dilution-based crystal violet microtiter plate biofilm assay was performed as described by Govantes [13] with modifications. Briefly, An inoculation culture was prepared as described above for the standard crystal violet assay. Then 3-fold serial dilutions of this culture were made to obtain series of wells containing decreasing amounts of inoculum. The microtiter plate was then processed and incubated as described above, and after 12 h of incubation the amounts of biofilm present in the wells were quantified by crystal violet staining as described above.

## 3 Results and discussion

### 3.1 Time-resolved and dilution-resolved crystal violet assays both display the dynamics of *P. aeruginosa* biofilm development and dispersal

**Fig. 2:**
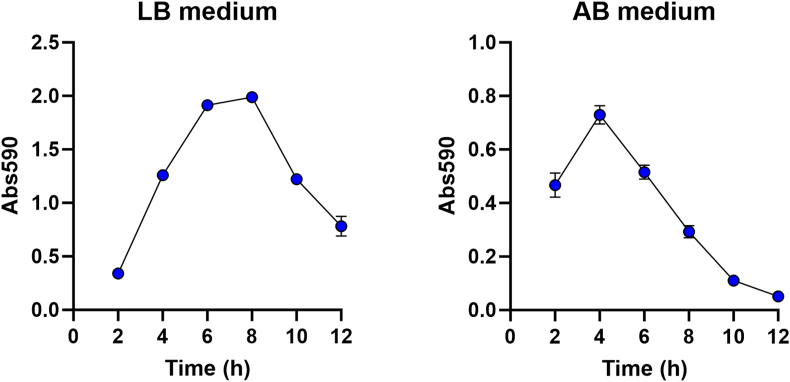
*P. aeruginosa* PAO1 biofilm formation in microtiter plate wells as a function of time. Replicate microtiter plates were inoculated with diluted (OD_600_ ∼ 0.1) *P. aeruginosa* cultures in LB medium (left) or AB medium (right), and after 2, 4, 6, 8, 10 and 12 h of incubation the amount of biofilm in the wells were quantified using crystal violet staining. The figure shows averages and standard deviations of three technical and three biological replicates. (For interpretation of the references to colour in this figure legend, the reader is referred to the Web version of this article.)

**Fig. 3:**
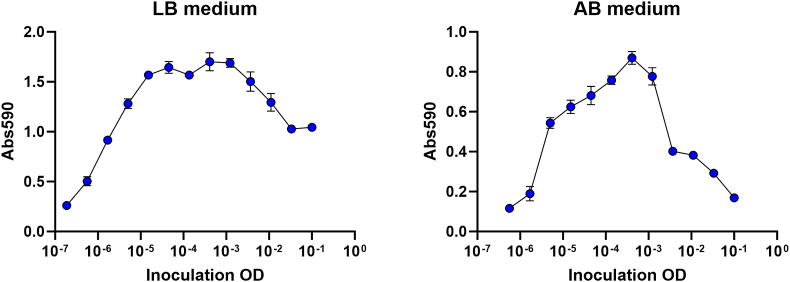
*P. aeruginosa* PAO1 biofilm formation in microtiter plate wells as a function of inoculum size. Microtiter plate wells were inoculated with serial diluted cultures in LB medium (left) or AB medium (right), and the biofilms in the wells were grown for 12 h after which they were quantified using crystal violet staining. The figure shows averages and standard deviations of three technical and three biological replicates. (For interpretation of the references to colour in this figure legend, the reader is referred to the Web version of this article.)

The serial dilution-based crystal violet microtiter plate biofilm assay was originally developed and characterized with *P. putida* as biofilm forming organism [12,13]. Here, we confirmed that the method is also applicable to one of the major biofilm-model organism, *P. aeruginosa*. We first assayed time-resolved biofilm formation, using replicate microtiter plates that were inoculated with a fixed inoculum size and incubated for various periods of time, after which the amount of biofilm in the wells was quantified by crystal violet staining. Fig. 2 shows the amount of biofilm as a function of incubation time, illustrating the buildup of biofilm followed by a decrease in biofilm biomass as the biofilms disperse. We then assayed dilution-resolved biofilm formation, using a microtiter plate that was inoculated with various inoculum sizes and incubated for a fixed amount of time, after which the amount of biofilm in the wells was quantified by crystal violet staining. Fig. 3 shows the amount of biofilm as a function of inoculum size. It is obvious that the curves obtained by the dilution-resolved technique are similar to the curves obtained with the much more laborious time-resolved experiments. Thus, the serial dilution-based crystal violet microtiter plate biofilm assay is ideal for direct and easy assessment of the dynamics of development and dispersal of biofilms.

### 3.2 Example illustrating the need to assess biofilm dynamics instead of quantifying biofilm biomass at a single time point

**Fig. 4:**
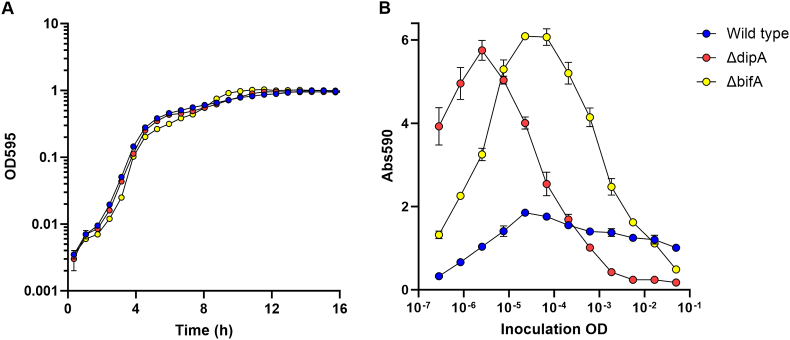
Kinetics of planktonic growth (**A**), and biofilm formation and dispersal (**B**) for the *P. aeruginosa* PAO1 wild type and isogenic *bifA* and *dipA* mutants. For the assessment of planktonic growth, microtiter plate wells were inoculated with diluted cultures of the wild type, *bifA* and *dipA* strains in LB medium, and optical density (OD_450_) was measured at regular time intervals using a Tecan microplate reader. For the assessment of biofilm growth, microtiter plate wells were inoculated with serial diluted cultures of the wild type, *bifA* and *dipA* strains in LB medium, and the biofilms in the wells were grown for 12 h after which they were quantified using crystal violet staining. The figure shows averages and standard deviations of two technical and three biological replicates. (For interpretation of the references to colour in this figure legend, the reader is referred to the Web version of this article.)

As an example illustrating the need to assess biofilm dynamics instead of quantifying biofilm at a single time point, we focused on mutants of different parts of the c-di-GMP signaling system in *P. aeruginosa.* The signal molecule c-di-GMP is a central regulator of biofilm formation in various bacteria, especially the Gram negatives [25]. C-di-GMP is formed through the activity of GGDEF domain-containing diguanylate cyclases (DGCs) that are capable of catalyzing the formation of c-di-GMP from two molecules of GTP, whereas degradation of c-di-GMP is caused by the activity of EAL- or HD-GYP domain-containing phosphodiesterases (PDEs). Most bacterial species possess multiple DGCs and PDEs, and evidence is emerging that specific output of distinct c-di-GMP pathways is achieved through signaling processes where specific DGC, PDE, and c-di-GMP effector/target components interact, or through the activation of c-di-GMP receptors with different affinities for c-di-GMP [26]. The annotated genome of *P. aeruginosa* PAO1 [20] contains 17 genes that encode proteins with a GGDEF domain, 8 genes that encode proteins with an EAL or HD-GYP domain, and 16 genes that encode proteins that carry both GGDEF and EAL or HD-GYP domains [27]. In our experiment, we used two different *P. aeruginosa* PDE mutants, one with a deletion of the *bifA* gene and one with a deletion of the *dipA* gene. The c-di-GMP phosphodiesterase activity of the BifA and DipA proteins has previously been demonstrated [[28], [29], [30]]. Initially we assessed the growth of the *bifA*, *dipA* and wild type strains in planktonic culture. As shown in Fig. 4A, the two PDE mutants grew with the same growth rate as the wild type in planktonic culture. We then determined the dynamics of biofilm development and dispersal for the three strains, using the serial dilution-based crystal violet microtiter plate biofilm assay (Fig. 4B). From this experiment we could conclude that the *bifA* and *dipA* mutants both formed more biofilm than the wild type (as expected), and the maximum amount of biofilm was about the same for the two PDE mutants. It was also clear that both PDE mutants are capable of biofilm dispersal when cultivated in the microtiter plates. However, the dynamics of biofilm formation and dispersal differed, with the *dipA* mutant forming biofilm faster and entering the dispersal phase earlier than the *bifA* mutant. Clearly, these conclusions would not have been evident if biofilm sampling was done at a single time point. The latter procedure would instead lead to misleading results depending on the time point chosen for sampling.

## 4 Concluding remarks

The crystal violet microtiter plate biofilm assay is often used to compare the amount of biofilm formed by a mutant versus wild-type or compound-treated biofilm versus non-treatment control. But due to the destructive nature of the assay it can only be used to quantify biofilm at a single time point. Accordingly, in many of the studies that have employed the assay, the amount of biofilm is assessed only at a single time point after a certain period of biofilm formation. However, if biofilm formation of a mutant or compound-treated biofilm occurs with different dynamics compared to that of the wild-type or non-treatment control, then biofilm quantification at a single time point may give misleading results. We recommend to use the serial dilution-based crystal violet microtiter plate biofilm assay for easy assessment of the dynamics of biofilm development and dispersal.

## CRediT authorship contribution statement

**Jens Bo Andersen:** Writing – review & editing, Methodology, Investigation, Formal analysis, Data curation, Conceptualization. **Morten Rybtke:** Writing – review & editing, Methodology, Investigation, Formal analysis, Data curation, Conceptualization. **Tim Tolker-Nielsen:** Writing – review & editing, Writing – original draft, Resources, Project administration, Funding acquisition, Formal analysis, Conceptualization.

## Declaration of competing interest

The authors declare the following financial interests/personal relationships which may be considered as potential competing interests:Tim Tolker-Nielsen reports financial support was provided by 10.13039/501100012331LEO Foundation. Tim Tolker-Nielsen reports a relationship with 10.13039/501100012331LEO Foundation that includes: funding grants. If there are other authors, they declare that they have no known competing financial interests or personal relationships that could have appeared to influence the work reported in this paper.

## Data Availability

Data will be made available on request.
